# Emotional Effects on University Choice Behavior: The Influence of Experienced Narrators and Their Characteristics

**DOI:** 10.3389/fpsyg.2016.00689

**Published:** 2016-05-24

**Authors:** Ana I. Callejas-Albiñana, Fernando E. Callejas-Albiñana, Isabel Martínez-Rodríguez

**Affiliations:** ^1^Department of Psychology, University of Castilla-La ManchaCiudad Real, Spain; ^2^Department of Spanish and International Economic, Econometrics and Economic History, University of Castilla-La ManchaCiudad Real, Spain

**Keywords:** emotions, informative video, consumption, university, narrative

## Abstract

This study analyzes the influence that experienced users of university resources might have as narrative sources of information for other students in the process of choosing their schools. Informative videos about the benefits of studying at the university provide a reference model. In these videos, a group of young people present their views and explain their reasons for choosing the university in which they are pursuing their degrees; the various narrators detail all the resources available. This study investigates whether the individual identifiers of these narrators (e.g., gender, age, physical appearance, nonverbal gestures such as smiling, posture) influence perceptions of the credibility of the information they provide. Among a sample of 150 students in their last year of pre-university training, the results demonstrate that the students' ability to identify with the narrators provides information and arouses emotions that inform their perceptions of reliability and therefore their consumption choices. None of these predictors appear to serve as determinants that can be generalized, but if emotional attitudes in response to narratives about the topic (i.e., the university) are positive, then they prompt a change in attitude toward that reference topic too.

## Introduction

The proliferation of private Spanish universities in recent years (of the 32 currently in operation, 14 are new since 2001), adding to the 50 public universities, all of which offer a wide range of degree programs, makes it imperative for these institutions to find new ways to attract consumers of their products, by encouraging diverse students to prefer their offerings to those provided in other, nearby regions. For example, the University of Castilla-La Mancha (UCLM) is a public university, located in the Secondary School of Spain, which manages to attract just 40% of the students in its surrounding community who qualify (i.e., those who pass an entrance exam). Yet data from the Ministry of Education, Culture, and Sports also reveal that UCLM includes students from other, nearby regions, such that overall, its average enrollment is comparable to that of other regions with similar characteristics.

These data in turn prompt an inevitable question: How do potential students in a particular region decide to study, or not, at the university located in their autonomous community? In its efforts to answer this question, and accordingly encourage more students to enroll, UCLM has undertaken a continual quest for improvement, seeking to advertise and diffuse its educational offerings, especially through online marketing strategies. For example, in online promotional videos, real UCLM students, graduates, or those who dream of attending the school describe their experiences at the university and their related views of its offerings. These videos then are available to be shared freely with potential enrollees. To test the effectiveness of this recruitment and marketing strategy, the current study seeks to analyze whether and how the narrators of these videos, who share their experiences with the product and thus provide signals regarding the prototypical users (i.e., students) of the university's offerings, affect the decision of students considering whether to enroll. In particular we consider whether the experienced narrators represent potential predictors of those decisions. To begin, we consider the different factors involved in this type of advertising and the reactions they might generate.

## Conceptual framework

The central variables underlying our empirical study reflect insights gained from research into electronic word of mouth (WOM), consumer behavior, and the relevance of emotions in decision making, as we review in this section.

### Electronic word of mouth

Advertising through various media formats represents a common strategy for online marketing. For example, informative videos on websites can facilitate users' access to pertinent knowledge, and they can reach these videos through their smartphones, computers, tablets, or other devices. Such videos also do not require information gatherers to read extended texts or rely on just still images.

These videos also can enable a strategy by which real people, who have personal experience with a particular product, appear in the videos to share their “word of mouth,” in which case it constitutes electronic word of mouth. According to the Association for Research of Media (2015), nearly 80% of Internet surfers read others' opinions or comments, and more than half of them express great confidence in this source of information. In particular, word of mouth affects 20–50% of all purchasing decisions (Marketing Directo, 2013). The increasing influence of eWOM suggests that it offers strongly compelling information to most consumers (Kozinets et al., 2010; Zhu and Zhang, 2010; Lim and Chung, 2011); it has long been well-established that the recommendations and opinions disseminated through word of mouth have far greater influence than other forms of paid advertising.

### Consumer behavior

Performance, from a psychological point of view, refers to the set of actions and behaviors that a subject exercises in response to external and internal stimuli. In a consumption context, this definition implies that consumer behavior is the set of actions conducted during a decision-making process, which arises due to some lack or perceived need. According to Wilkie (1996), consumer behavior involves the physical, mental, and emotional activities that people adopt to access the products and services they believe will meet their needs and desires. Arellano (2002) states that consumer behavior is related to the internal or external activity of the individual or group of individuals aimed at meeting their needs by purchasing goods or services.

Marketing researchers should be aware that there are many external variables that influence buying behavior (Rivas and Grande, 2004). To unify the various proposals we have to focus on connotations related to the common elements that can be seen in all of them, such as:

- A set of actions serve to pursue an end, following the detection of a need or some precise motivation, such that the actions are clearly intended to meet that need (e.g., product purchase).
- The presentation of a product can generate an attraction.
- Several factors influence the decision process, including cultural, social, psychological, and personal factors.

However, many decisions rely on quick thinking or emotions, such that they are relatively unconscious. Decision makers do not always undergo a rational process to arrive at conscious decisions. In some cases, others with some level of expertise or experience can prompt certain consumer behaviors, especially if they highlight needs that become personal aspirations. This effect implies a process of motivation, which refers to “the intensity, direction, and persistence of the effort to achieve a goal” (Robbins, 2004, p. 156).

Furthermore, two main streams of prior research identify the key predictors of consumer behavior. On the one hand, some studies focus on how consumers receive information and process it for use, premised on the notion of a fundamentally rational consumer (Solomon, 1998), who focuses on the decision process and brand evaluation. This stream of research primarily uses cognitive psychology as a foundation. On the other hand, other research notes the emotional, social, and cultural aspects of consumer behavior and therefore centers mostly on investigations of feelings and emotions. These two streams actually are complementary, in that they cover the various reasons people engage in the act of buying, and they span multiple cause-and-effect rationales for human behavior. As Bagozzi et al. (1999) suggest, a necessary condition for an emotional response to a situation or event is that the person already has some personal interest in the situation and is willing and able to assess the situation to support or mitigate this sense of interest or concern.

Focusing on the decision process involved in choosing a University, different stages of a clearly complex decision were analyzed, for example Chapman (1986) establishes five stages in a model of the college selection process: 1- Pre-search behavior, 2- Search behavior, 3- Application decision, 4- Choice decision, 5- Matriculation decision.

Another analysis of the stages of University choice is that made by Kotler and Keller (2009) establishing 5 such stages: 1 -Need arousal, 2- information search, 3- alternative evaluation, 4-decision, 5- decision implementation, 6- post- purchase evaluation. The first three involve a search for a system of prior assessment based on the motivation and characteristics of universities that have previously been selected by the subject, including those given by word of mouth, or other factors that may be attractive, all of them leading to the establishment of criteria for optimal evaluation in the final decision (stage five), although the process does not end with the deployment option but rather advances toward the final phase (6), which involves evaluation of the success of the choice and the degree of satisfaction.

Using this as a reference we can emphasize that universities should establish attractive offers and maintain established services to the standard expected by its users.

We now analyze models of University choice based on factors that promote the final decision. Chapman (1981) performs a study about relatively fixed university in attracting students. Chapman describes two types of factors related to the choice of college students, based on variables such as skills and prerequisites of students and the student's choice of college, highlighting three types of variables based on external influences, relevant people within the family environment, friends or teachers; University features (localization, co-variation of degrees), and college efforts to communicate with student (written information, campus visit, admission).

Raposo and Alves (2007) establish a classification of models based on factors that influence the choice process of a college or university:

- Models based on economic cost and profitability are rational in character as they focus on the evaluation of factors such as: academic reputation, academic accreditations, costs, job prospects, teaching quality, variety of education, financial aid (Chapman, 1993; Coccari and Javalgi, 1995; Kallio, 1995; Holdsworth and Nind, 2005).
- Models based on socio-emotional aspects of character mainly value personal and social cost, and assess factors such as personal contacts, influence of parents, location, social life, availability of a variety of degrees (Donnellan, 2002).
- Mixed models that combine and give the same weight to economic cost and staff (Murphy, 1981; Lin, 1997; Soutar and Turner, 2002; Shanka et al., 2005).

Following the exploratory study by Raposo and Alves (2007) whose claim is based on establishing a model of University choice that brings together all criteria derived from the models listed above in four dimensions: institution overall reputation, educational offer, previous knowledge about the institution, individual factors, influence of others. The results show the difficulty in determining generalizable and stable criterion factors for all persons and areas of knowledge as there is a significant connection between all these factors, but it is not decisive, although highly influential variables stand out as markers of choice of University: proximity to home, costs, parents, and secondary school teacher recommendations, etc. It nevertheless provides interesting data regarding the effort universities must make to attract students because it shows the need to address all dimensions referenced to the same degree of importance, in order to ensure that the choices students make will be based on one of these factors.

In our study, one of the objectives is based on the dimension of prior knowledge and the relationship with individual and influence of other factors, and promotion of institutions as prerequisites to then move into one of the modes of uptake data: the promotional video.

### Emotions in advertising

Advertising uses various creative ways to win over consumers. Emotional advertising is an effective form of communication that companies can use to achieve differentiation from competitors, because this format awakens diverse sensations among audiences. (López, 2007). For example, The ARS Group (2010) uses the pleasure–arousal–dominance (PAD) theory to explain the role of emotion in advertising and how it generates preferences, sales, and market share. The Group has developed a technique to measure emotion and nonverbal responses, using visual techniques. The approach has been used successfully in more than 600 proprietary studies and 35 academic studies around the world. It also aligns with theory, as proposed by Gardner (1983) and subsequent (Clark and Isen, 1982; Burke and Edell, 1989) studies, that suggests that the affective responses influence cognitive processes, including evaluation, memory, and judgment.

Because so many decisions are subconscious, marketing strategies generally aim to provoke positive expectations among consumers, such as by forging positive emotions that evoke pleasant memories and benefits related to the consumption object. Emotional marketing acknowledges that emotions can satisfy consumers while also creating expectations about their feelings and sensations. To ensure that emotional marketing leads to consumption behaviors, marketers must establish a series of steps, such as identifying the wants and needs of consumers, establishing a link between their interests and intangible properties of the product, and creating a communication strategy that expresses the identified emotional perceptions, such that that both motivation and emotional processes influence consumers' psychic activity (Fernández-Abascal, 1997).

The basic process of communication theory emphasizes the importance of the source (sender of message) as persuasive element and the characteristics to be taken into account such as credibility, attractiveness, similarity, familiarity, and reputation. The most important is credibility, which depends on two fundamental factors: competence, understood as experience or reliable information which informs the skill in handling the transmitted message fluently (Briñol et al., 2001), and the sincerity that should allow the message to be sent with no profit motive and a lack of persuasive intent (Morales et al., 1997).

The attractiveness of the sender is one of the most persuasive elements in purchaser choice as it causes more attention to the message given, it influences the identification process because they may want to act the same way, and it can increase credibility by providing greater communication skills (Chaiken and Eagly, 1983). Furthermore, this attractiveness can be a critical component in making the message more effective (Schlecht, 2003).

However, the effects of source attractiveness can be overcome by the effects of credibility so that a highly credible source emitter with low appeal can be more effective than another with high attractiveness and low credibility (Wachtler and Counselman, 1981).

The credibility model of Ohanian (1990) relates these elements, indicating that purchase intention may be affected by the perceived attractiveness, reliability and level of experience of the source, draw attention to the positive characteristics of the communicator, affecting the acceptance of the message by the receiver.

The perceived similarity of the source by the receiver is also important especially if it refers to the same social group and provides attitudinal similarity (Briñol et al., 2001).

Both familiarity and reputation can be persuasive factors provided they do not become habitual, i.e., they change both the characters and the message (Briñol et al., 2004).

The choice of message transmission is one of the most interesting points in the process of persuading someone to purchase a product. The choices are to show directly, or to use a spokesperson or celebrity; however, in both cases the above factors should be taken into account. The study by Sertoglu et al. (2014) measures the differences in credibility perceived in Turkish society regarding the use of spokesman and celebrities and their impact on the purchase decision, obtaining that spokespersons are perceived as a more reliable and positive influence on purchase intent that celebrities, whereas celebrities are perceived as more attractive. This is in agreement with the studies of Van der Waldt et al. (2009). However, the society being studied should be considered when generalizing the results.

Long studies on the involvement of emotions in the process of purchasing decision have great relevance and Zajonc and Markus (1982) point out that there is a significant relationship between affective and cognitive reactions that affect the formation of the attitudes.

The theory of cognitive assessment studies the impact of emotions on behavior based on the cognitive assessment of the subject toward a certain behavior. According to Arnold (1970) initial evaluations begin the emotional sequence and arouse both appropriate actions and emotional experience, so that physiological changes accompany actions and experiences but do not start them. Lazarus (Lazarus, 1991) consider that the nature of the assessment of cognitions underlying emotional reactions is related, firstly, with a primary assessment linked to the importance of the event at the organic level, and then the organism's ability to face the consequences of the action should be assessed. However, Taylor (2009), believes that emotions can exist throughout the decision process with cognition.

The regulatory role of conduct in relation to behavior is also determined by the prospect of the cognitive assessment process which leads to emotional experience as the level of arousal. Di Muro and Murray (2012) examine how the interaction between the level of excitement and the valence of the emotional state affects consumer preferences, so that a positive mood tends to the choice of products according to their level of arousal, while a negative mood will choose products unsuited to the current level of excitement. Research Carrera and Oceja (2007) indicate that level of emotional intelligence is significant for handling negative emotions in the selection process as it can achieve positive results.

The impact of emotions on decisions related to consumption has acquired great significance today (Kotler et al., 2010; Peter and Olson, 2010), which leads to the need to develop marketing strategies based on the consumer rather than holistic strategies.

Finally, the new discipline of neuromarketing is based on the study of the brain in an effort to understand unconscious patterns that govern the buying process. Researchers using these techniques argue that consumers' attention can be captured through by images that excite them, not by rational argument. The more intensely deeper emotions are generated, the better the neurological brain connection encouraged by consumer advertising can reinforce these neural networks. We can point to many examples of the application of this notion, such as olfactory perceptions prompted by the smell of perfumes or cakes in bakeries, auditory perceptions sparked by contemporary music played in stores that target young consumers, or visual perceptions resulting from lighting that emphasizes a particular product. This study seeks to identify relevant variables in promotional videos that evoke emotional connotations that also can predict the consumption decisions among their audiences.

## Research

Concerning the information about the ethics approval, we would like to highlight that: We always give participants detailed and written information about the study and the procedure. We do not collect data related (direct or indirectly) to the subject health at all and usually do not refer to the declaration of Helsinki when informing the subjects. We always guarantee the anonymity of the data collected. Consent was implied by voluntary completion of the questionnaire.

Several research goals drive this work. In particular, we seek to:

- Demonstrate preferences for eWOM information about a product.
- Establish the relationships between the characteristics of the narrators, or senders of eWOM, with study participants, or eWOM recipients.
- Check for predictive variables related to the demographic characteristics of the narrators in the informative UCLM video.
- Analyze participants' intentions to study at UCLM after viewing the video and narrators.

To achieve these research goals, this study uses six independent variables related to the study participants (i.e., potential students at UCLM) age (16–18 years), gender, high school course (controlled for, second baccalaureate^1^), residence (i.e., city with < 10,000 inhabitants, city with more than provincial capital), course of prior studies.

High school specialty (science, humanities/social sciences, or arts), areas of University knowledge, arts and humanities, social sciences, health sciences, social/legal sciences, or engineering and architecture). The dependent variables describe the character of the narrator in the video. They include the narrator's age (15–30 years), gender, and degree.

Sample of secondary schools in Ciudad Real province, we used stratified random sampling, based on the number of inhabitants. Accordingly, we defined three groupings based on residence, reflecting smaller cities, larger cities, and the capitol. We randomly sampled people who lived in all three locations. Localities included secondary schools: 16 in smaller cities (< 10,000 inhabitants), 12 in larger cities (>10,000 inhabitants), and 1 in the capitol. The random draw then led us to select populations from Argamasilla de Alba (7201 inhabitants), Puertollano (50,608 inhabitants, seven centers of which randomly chooses one of them), and Ciudad Real (74,960 inhabitants). This distribution produced the sample detailed in Table 1. However, the information in Table 1 does not include the type of bachelor's degree, because this option is not available to customers in any of the selected Secondary Schools, and one of the preconditions for sample participants was that they were attending their last year of their baccalaureate degree.

Table 1**Narrator traits**.

**Table:** 

**No**	**Name**	**Age**	**Slogan**	**Degree**	**Branch (1, H&A; 2, Sc; 3, H; 4, SS&L; 5, E&A)**	**Situation (1, Final/post; 2, Actual; 3, Future)**
1	David	26	Entrepreneurial culture	History	1	1
2	Josete	22	Passion for heritage	Humanities	1	2
3	Juan Miguel	16	From the classroom to the field	Agricultural Ing.	5	3
4	José	28	The decision that changed my life	Law	4	1
5	Ana	29	Dream fulfilled	Medicine	3	1
6	Pablo	25	Double degree in Law and Business Administration	Law-BA	4	2
7	José Manuel	30	Industrial engineer and guitarist	Engineering	5	1
8	Esther Mariano			Bussines Administration	4	2
9	Rocío	27	Monitoring the Future	Environment	2	1
10	Daniel	23	Artistic vocation	BBAA	1	2
11	Lucia	15	Artistic vocation	BBAA	1	3
12	Christian	26	Toledo to heaven	Sport Sc	4	1
	SUMMARY				H&A = 4; Sc = 1; H = 1; Ss&Ls = 3; E&A = 2	Final/post 6; Actual = 3; Future = 2

*A&H, Arts and Humanities; Sc, Sciences; H, Health Sciences; SS&L, Social and Legal Sciences; E&A, Engineering and Architecture*.

**Table:** 

**SAMPLE**								
	**Sex**		**Age**		**High school specialty**			**Total**
	**Men**	**Women**	**16**	**17**	**18**	**Sciences**	**Humanities**	
Stratum 1	11	20	0	28	3	24	7	31
Stratum 2	13	50	1	60	2	49	14	63
Stratum 3	21	35	1	54	1	35	21	56
Total	45	105	2	144	6	108	42	150

Complete a questionnaire to provide the data for our analysis. We solicited cooperation from the teachers, who agreed to devote some class time to allowing the students to complete the questionnaires, and the schools which made computers available for the students to complete the surveys. All schools had dedicated computer rooms or spaces, so this requirement was not a limitation.

The questionnaire (see the Appendix) consisted of two parts: a first part to collect demographic data about the participants and a second part that required participants to visit the website of the University of Castilla-La Mancha and locate a banner entitled “ASI all started,” which linked to the informative video. The video featured 12 narrators, describing their first-hand relationship with UCLM from different perspectives, representing different campuses, degrees, and levels (undergraduate, graduate), as well as some people who simply expressed their interest in studying at the university. Table 1 summarizes their characteristic features. On the website, participants also could access more information about each narrator by clicking on a related title, which revealed that person's history. Each participant could choose to read or listen to the personal information. After receiving these details, the participants completed several items related to their resulting impressions. Requested issues are: (1) which character you've chosen? (2) Why did you choose? (3) How old you are or think has the character you've chosen? (4) What do you like best of what it has been told? Why? (5) What it is the least liked you? Why? (6) It has been clearly expressed? Highlights some phrases or comments that have caught your attention; (7) Indicates aspects of his gestures, look, smile, posture, physical appearance; (8) It has shed new light on the University of Castilla La Mancha; (9) has your opinion changed about making your studies at this University?

Ultimately the idea is to confirm whether the information received from agents that represent similar characteristics or an expectation can be highly efficient for the choice of university studies. The work of Hung and Mukhopadhyay (2012) examines how visual perspectives taken to assess a situation are related to seeing themselves as actors in that situation influencing the intensity of the experience (related to the characters in the video).

The focus is on identifying the factors that are decisive in the choice of University and the motivation behind them, for example Keshishian and McGarr (2012) note that, for New York students, intrinsic motivation, social relationship, and the possibility of achieving the goal set by the student, act as important predictors and may be helpful when preparing recruitment strategies. However Mohamad et al. (2012) found that for Malaysian students, the fees (academic cost) were the most representative factor when choosing university.

Another notable factor is that the protagonists are natural storytellers, not celebrities. The fact that it is the image and the slogan which decide the choice or otherwise of the character is due to the claim that the greater personal involvement, the greater the tendency to pay attention to the message, to the arguments and not to peripheral signals such as the attractiveness of the source (Morales et al., 1997).

Gender differences regarding the choice of the character are also a differentiating factor when choosing and defining information about search behavior as a criterion for guidance through the media (Barber et al., 2009; Meyers-Levy and Sternthal, 1991).

In these terms, we want to know what the relevant factors for students in Castilla La Mancha (Spain) would be, and whether the marketing strategy is suitable or new methods may be found.

## Results

The data analysis relied on the software package SPSS, version 22. Our analysis revealed insights that reflected our research objectives. First, the participants clearly preferred eWOM, such that all of them (*n* = 150, 100%) chose to watch the video of the narrator versus reading their history.

These results are consistent with related studies by different authors (Chaiken and Eagly, 1983; Meyrowitz, 1985) on preference in video or audio media over printed sources.

Second, to investigate the links among the study variables, we performed a preliminary analysis of the frequencies of the characteristics of the narrators, to test its effect on the participants' choices. With the significance of the variables and their χ^2^-values, we built a cross-contingency table related to the features of the narrators and the personal characteristics of the participants. Then, we used a logistic regression to determine the predictive ability of these variables for participants' choice to study at UCLM.

Third, we consider the frequencies of the variables related to personal characteristics: age, gender, course of studies, situation, and narrator chosen. These descriptive statistics, with the exception of the age variable, are categorical variables. Therefore, it is important to acknowledge the descriptive statistics (Mo), or the value with the greatest frequency distribution data, without ignoring that categorical variables are inherently encoded. We find that the age of the narrator that earns the highest percentage rating is 30 years (29%); ages older than 25 years account for 80% of the total (Table 2).

**Table 2:** **Frequency of the age of the narrator**.

**Age**	**Frequency**	**Percentage**	**Descriptive statistics**
15	1	7	M 26.96
22	15	9.9	27.00 MB
23	4	2.6	MDN 30
25	9	6	*SD* 2583
26	27	17.9	6670 Variance
27	26	17.2	
28	2.3	15.2	
29	16	10.6	
30	29	19.2	

However, the character most frequently chosen has the highest age (30 years), followed by 28, 26, and 23 years, so it is understood that the preference is in the expectation of achievement, and that in the attractiveness of the slogan in these cases the coincidence is in the mix of interests, not only academic but also personal (see “character traits”).

In addition, as we show in Table 3, male narrators are more likely to be selected than female narrators. The number of male narrators in the video was greater, by more than 50%, though this statement does not need to be relevant. However, we also uncover an apparently inverse relationship with regard to gender: Greater percentages of female (*n* = 105) and male (*n* = 49) participants in the sample did not choose a narrator of the same sex. With regard to the course of study, the most mentioned focus of the narrators was social and legal sciences (37%, Table 4). Regarding the focus of the participants in their baccalaureate studies (science or humanities), somewhat surprisingly, more students had chosen to study science (*n* = 108). Even if we include arts and humanities, the percentage value for science remains greater. This finding makes more sense in relation to the findings regarding their planned university studies, as we discuss subsequently. Furthermore, as Table 5 shows, the participants preferred narratives by people who currently are students at the university (80.9%), followed in much smaller percentages by students who had completed their undergraduate degrees and were continuing on to postgraduate studies (17.9%), and then by other prospective students (0.7%).

**Table 3:** **Frequency of the gender of the narrator**.

**Sex**	**Frequency**	**Percentage**	**Descriptive statistics**
Male	107	70.9	Mo 1
2 = Female	43	28.5	

**Table 4:** **Frequency of the knowledge of the hero Rama**.

**Discipline**	**Frequency**	**Percentage**	**Descriptive**
			**statistics**
1 = Arts and humanities	24	15.9	Mo 4
2 = Sciences	26	17.2	
3 = Health sciences	16	10.6	
4 = Social s. and legal	57	37.7	
5 = Engineering and architecture	27	17.9	

**Table 5:** **Frequency of situation of the protagonist**.

**Situation**	**Frequency**	**Percentage**	**Descriptive statistics**
1 = Finalizado- Post	122	80.8	1.00 MB
2 = Current	27	17.9	
3 = Future	1	7	

Table 6 contains the frequency of selections of narrators representing different fields of study. 30-year-old engineer is engaged in his postgraduate studies. Thus, he effectively represents our findings regarding the most preferred gender, age, and course of studies. However, his degree, engineering, represented only the penultimate position in terms of choices. The next most frequently chosen narrator was 27-year-old Rocio, a student of environmental science, who also represents the most preferred features in terms of age. In contrast, two narrators did not prompt any significant selections. Both of them were younger than 25 years of age and did not currently attend UCLM but instead described their desires to go there (i.e., Juan Miguel and Lucia).

**Table 6:** **Frequency of choice of the protagonists**.

**Protagonists**	**Frequency**	**Percentage**	**Descriptive statistics**
1 = DAVID	4	2.6	Mo 7
2 = JOSETE	15	9.9	
3 = JUAN MIGUEL	0	0	
4 = JOSÉ	23	15.2	
5 = ANA	16	10.6	
6 = PABLO	9	6.0	
7 = JOSÉ MANUEL	29	19.3	
8 = ESTHER Y MARIANO	0	0	
9 = ROCÍO	26	17.2	
10 = DANIEL	4	2.6	
11 = LUCIA	1	0.7	
12 = CHRISTIAN	23	15.2	

### Cross-table analysis

Fourth, the cross-tabular analysis details the relationships across variables. A relationship between two categorical variables cannot be established simply by observing the percentage frequencies; rather, we required some measure of the association and its corresponding significance. Therefore, working with statistical non-parametric tests, we relied on Pearson chi-square (χ^2^) values to determine whether a relationship exists between two categorical variables, reflecting the difference between the expected and observed frequencies. For the hypotheses tests, we note the degree of significance, which indicates the certainty associated with the relationship, though not of its strength. For that measure, we turn to the V Cramer and Pearson Phi, which indicate relationship intensity. The contingency tables accordingly present the independent categorical variables in rows and the categorical dependent variables in the columns. Using the statistical χ^2^ measure also requires that the expected frequencies are not too small. That is, if frequencies of < 5 are expected, they must not exceed 20% of all expected frequencies. If the percentage exceeds 20%, then we must interpret the Pearson statistic with particular care. In Table 7, we denote these assumptions with an asterisk. Finally, the university course chosen by the participants was not estimated, because it remains a constant factor for these baccalaureate students.

**Table 7:** **Contingencies**.

**Character**	***N* = 150**	**χ^2^**	**gl**	**Sig asymptotic (2-sided)**	**C**	**Phi**	**V Cramer**
SEX	Age	0.526	2	769	0.059	0.059	0.059
	Sex	7369	1	0.007	0.007	0.222	0.222
	High school specialty	35.912	1	0	0.44	−0.489	0.489
	Secondary school	13,629	2	0.001	0.286	0.301	0.301
	Degree	45.789^*^	6	000^*^	0.484	0.553	0.553
	Study at UCLM	1898	1	0.168	0.112	0.112	0.112
AGE	Age	31.351^*^	16	0.012	0.416	−0.457	0.323
	Sex	13,441	8	0.98	0.287	0.299	0.299
	High school specialty	78.101	8	000^*^	0.585	0.722	0.722
	Secondary school	78,671	3	000^*^	0.587	0.724	0.514
	Degree	129.839	48	000^*^	0.681	0.931	0.38
	Study at UCLM	23.061	8	0.003^*^	0.365	0.392	0.392
BRANCH	Age	6134	8	0.632	0.198	202	0.202
	Sex	9767	4	0.045	0.247	0.255	0.255
	High school specialty	39,876	4	000^*^	0.485	0.516	0.516
	Secondary school	62.608	8	000^*^	0.543	0.646	0.457
	Degree	81.082	24	000^*^	0.592	0.735	0.368
	Study at UCLM	16,077	4	0.003	0.311	0.327	0.327
SITUATION	Age	24,620	4	0	0.375	0.405	0.286
	Sex	0.491	2	782	0.057	0.057	0.057
	High school specialty	7.222	2	0.027	0.214	0.219	0.219
	Secondary school	16,830	4	0.002^^*^^	0.318	0.335	0.237
	Degree	31,137	12	0.002	0.415	0.456	0.322
	Study at UCLM	1445	2	0.485	0.098	0.098	0.098
CHARACTER	Age	32.022	18	0.022	0.419	0.462	0.327
	Sex	14,248	9	0.114	0.295	0.308	0.308
	High school specialty	88.04	9	0	608	0.766	0.766
	Secondary school	86.556	18	0	0.605	760	0.537
	Degree	142.049	54	0	697	973	0.397
	Study at UCLM	25.667	9	0.002	0.382	0.414	0.414

* *Expected frequencies under 5 to over 20%*.

To interpret the data in Table 6, we use reference variables, associated with each narrator feature and its association with the participants' characteristics. For example, the gender of the narrator had no association with age. Nor did we find any effect of the narrator's gender on the choice to study at UCLM. The correlations of the genders of the narrators and the participants also revealed no evident relationship [χ^2^ = 7360 (1, *N* = 150) = 0.007, *p* > 0.05]. Of the 49 male respondents, 42 chose a narrator of the same sex, and 7 chose a female narrator. However of the 107 women surveyed, 65 chose men and 36 women, such that more than 60% of these participants opted for narrators of the opposite sex. In addition, the type of facility is significant [χ^2^ = 13,629 (2, *N* = 150) = 0.00, *p* < 0.05], and the degree of intensity of.301 *V Cramer* indicates a moderate relationship. The type of baccalaureate [χ^2^ = 35,912 (1, *N* = 150) = 0.00, *p* < 0.05] is statistically significant, but the intensity ratio (*V*. indicates a strong degree of moderate intensity). The relationship, according to the ratio phi = −0.489, also is reversed, such that the variables fluctuate and when one increases, the other decreases in value. The type of area of university knowledge future of the students surveyed, in relation to the gender of the narrators, indicates that χ^2^ = 45,789 (6, *N* = 150, *p* < 0.05), but 21.4% count < 5, so the significance of these variables is not sufficient.

The age of the narrator relates significantly to several other variables (high school specialty, secondary school, areas of university knowledge, study at UCLM) but cannot be regarded as a reliable predictor, because the frequencies of < 5 exceed 20%. The age and gender of the participants offer no significant results.

The field of study that the narrator is pursuing correlates positively with the gender of the participants [χ^2^ = 9767 (4, *N* = 150) = *p* < 0.05], but the intensity *Cramer V* = 0.045 indicates a discreet relationship. The type of baccalaureate degree and the narrator's field of study showed a positive [χ^2^ = 38.879 (4, *N* = 150) = *p* < 0.05] and intense (*Cramer V* = 0.516) correlation. The degree also relates related to the study at UCLM [χ^2^ = 16,077 (4, *N* = 150) = *p* < 0.05], with a relatively high intensity. The remaining variables have no influence. Regarding the situation of the character, not influence results are statistically significant in the variables analyzed regarding subjects surveyed.

Although we have presented the cross-table results for the narrators, it was expected that the results were not reliable regarding expected frequencies as they have been fractionating in features that they can be representative (i.e., age, sex, type of area of knowledge of university qualifications, etc.).

Study pertains to the relationship between the variables reflecting the intended choice to study at UCLM or not. The contingency tables between choosing UCLM or not and other student characteristics (age, gender, high school specialty, secondary school, area of university knowledge future) indicate significant relationships only between the choice of UCLM and the type of school (Table 8), which in turn reflects the number of inhabitants in the region. Starting with the frequency determination, we find that 55.3% of participants indicated that they would not chose to study at the UCLM, whereas 44.7% prefer to study there, though men more frequently indicated that they would not choose to study at UCLM (61.2%) than did women (52.5%). The statistical results also indicate a χ^2^-value of 10,848 [(2, *N* = 150) = *p* = 0.004]; moderate relationship between the two variables. Ultimately these data appear to exert significant influences on Secondary School in the choice variable UCLM. This relationship is reflected in the percentages of frequency expressed: students from towns with fewer than 10,000 inhabitants are more likely not to choose UCLM as the location for their higher education studies.

**Table 8:** Cross-Tabulation UCLM * Secondary School.

		**CENTER**		
		**Less than 10,000**	**More than 10,000**	**Capital**
UCLM	NOT	25	33	25
	YES	6	30	31

### Logistic regression analysis

The logistic regression model seeks to predict the estimated probability of a dependent variable and present some possible values (1 = yes, 0 = no), depending on the different values for the independent variables. That is, it reveals how dependent variable relates to one or more independent variables multinomial logistic regression and categorical variables that are not dichotomous, so that we can classify the participants according to the values of a set of predictors. Because our research goal is to analyze the effectiveness of a promotional device, according to factors involved in it, we define the variable Y as interest in studying at UCLM or not and variables X as the gender of the narrator (GENDERNA), the narrator's areas of university knowledge (AREANA), and the environment of secondary school (SECONDSCHO). These X variables thus reflect the results of the observed factorial relationship variables. The test of the predictive nature of this model depends on the extent to which the logarithm of the odds ratio increases when we observe the presence of these variables.

To test this relationship between predictor variables has made an analysis of the Hosmer-Lemeshow (B) to evaluate the goodness of fit and check for predictive relationship between selected as independent and dependent variables, and the Wald test (W) to check the plausibility of the model.

Table 9 contains the estimates of the parameters for the final model of METHOD = ENTER. SEXOPE. RAMAPE. CENTER. The results reveal that χ^2^ = 26,356 [(7, *N* = 150) =, *p* < 0.00]. The lack of consistency of the predictive model means that we cannot establish its significance, despite some suggestion that the key character is a woman with some degree of knowledge about science.

**Table 9:** **Estimates of the model**.

**UCLM ^a^**		**B**	**Standard Error**	**Wald**	**gl**	**Sig**.	**Exp (B)**	**95% confidence interval for exp (B)**	
								**Lower limit**	**Upper limit**
**METHOD = ENTER. GENDERNA-AREANA-HIGH SCHOOL**									
DO NOT	It intercept	18.610	783	564.71	1	000			
	[CENTER = 1]	1582	612	6690	1	010	4863	1467	16,121
	[CENTER = 2]	−089	414	046	1	830	915	406	2060
	[CENTER = 3]	0^b^			0				
	[SEXOPE = 0]	−18.490	880	441.21	1	000	9333E-	1662E-9	5240E-8
	[SEXOPE = 1]	0^b^	.	.	0				
	[RAMAPE = 1]	−301	683	195	1	659	740	194	2821
	[RAMAPE = 2]	−17.906	903	392.96	1	000	1673E-	2849E-9	9827E-8
	[RAMAPE = 3]	−20.696	000		1		1027E-	1027E-9	1027E-9
	[RAMAPE = 4]	−083	514	026	1	872	920	336	2522
	[RAMAPE = 5]	0^b^			0				

a *The category of reference is: YES*.

b *Set to 0*.

### Analysis of open questions

By studying participants' responses to the open questions in the questionnaire, we can address some alternative explanations and test some related concepts. In particular, we gauge the participants' first impressions of the narrators, their perceptions of the narrators' communicative ability, and their physical traits. Then we investigate if any of these perception change their view of the information received about UCLM idea and their choice of whether to study at UCLM or not. In this section, we present the most relevant findings, in the order in which they appeared in the questionnaire (see the Appendix).

- *Knowledge of UCLM*. More than 80% knew that UCLM is divided into multiple campuses; 60% that is published in official website of University (www.uclm.es) and about 40% know that they could pursue various degrees, but 10% claim to know nothing about the university.
- *Liking of the narrator*. Most participants highlighted the narrators' stories as interesting and expressed high valuation of the information transferred about university teachers, peers, and resources. A high percentage of participants did not indicate any negative aspects and indicated at least some identification with the narrators.
- *Verbal expression*. The narrators presented their stories clearly, without the use of rhetorical or technical language, according to the participants. These participants also noted the presence of statements related to a desire for achievement or fulfillment of dreams.
- *Gestural expression and physical traits*. Some notable gestures that the participants regarded positively included security, tranquility, a sincere smile, and personal proximity.
- *Causes of the choice to attend UCLM or not*. Many participants did not want to study at UCLM because they did not want to leave their region or because it did not offer the degree they sought. Those who chose to study there, in parallel, often cited its proximity and offering of their desired degree.

Despite acknowledging that they received broad, attractive information about the UCLM, 68.2% of the participants noted that they did not change their initial intentions with regard to choosing the UCLM for their higher education; others often expressed basic doubt in categorical responses, such as “I don't know. Maybe. I'll think about it.”

## Discussion and conclusions

The study data reveal that the promotional video for UCLM meets the standards for a marketing strategy that leverages eWOM, in both format and the argument set out, and it helped increase positive attitudes among the audience who read or heard the stories of the different narrators. These results emphasize the influence of the communication channel in decisions and persuasive capacity. Through the video as a resource, the message becomes visually important, is expressive and encouraging direct printing and images, facilitating perceptions of more intimate relationships with the characters (Meyrowitz, 1985). These reactions immediately follow the viewing of the information through the source, since the actual decision is often not present to act as an explicit signal for consumer choice (Monroe and Chapman, 1987).

In short, positive attitudes toward the decision to study at UCLM increased after viewing, although this does not ensure that the time that elapses until the actual decision cannot vary based on perceptual factors and the magnitude of emotional responses (Meyers-Levy and Sternthal, 1991).

Our findings also reveal some links across the variables studied. Considering the demographic data (age, gender, degree), it seems logical to predict that participants would prefer to receive eWOM from narrators whose characteristics are similar to their own. However, we determined that female participants chose more narrators of the opposite sex, whereas men chose same-gender narrators.

Although the literature based on the difference in information sources between men and women warns that the discrepancy is related to interests or knowledge (Holbrook, 1986; Fischer and Arnold, 1994), it is possible to explain it in terms of differences in information processing (Meyers-Levy, 1989). Women are more thorough when coding nonverbal messages (Everhart et al., 2001), they are also more affected by visual stimuli and are more emotional (Dittmar et al., 2004). Men are more analytical and therefore more objective (Kim et al., 2007).

We must not forget that in our study, the number of male narrators is greater than that of women as an influencing factor when choosing, as are characteristics related to the source, mentioned above, and the prior credibility of the character to the argument.

A similar result applies to the choice of degree, which revealed an effect not of the similarity of the degree or the focal field of study that the participants aspired to but rather the influence of the suggestive content of the slogan used to attract their attention to certain stories—namely, those involving personal achievement, especially in relation to art and engineering studies that might lead to the fulfillment of a dream. The participants also preferred stories told about the present or past, reflecting lived experiences or experiences in progress. In summary, the interest in the product (university) is most closely linked to experienced users, who continue to study, who are men, and whose narratives suggest a history of achievement, which might be related to their future aspirations as academics. In this point, gender differences can also be interpreted. Men are less likely to get involved in the stories, but women tend to enter into the story from personal experience (Kim et al., 2007).

However, we cannot establish any predictive traits of the narrators, such that we could anticipate which of them lead audiences to choose UCLM.

This study also helps identify some possible causes of “leakage” of students to other universities. The results indicate that the most significant cause is due to location factors, reflecting both the actual region and the size of their home towns. For example, people from smaller cities appear most interested in “flight” from their location region.

In short, the use of the promotional video to share information about UCLM is effective, if discreet, and provides a resource that can capture the attention and spark positive attitudes; in no case did it generate negative reactions (e.g., putting positive intentions to choose UCLM in doubt). To advance and continue this investigation, it would be interesting to track the students who participated in this study to specify their final decision. Because these students often are the consumers but not necessarily the buyers of the university product, it also would be interesting to determine if the preferences and choices of parents (buyers) match those of the students (consumers).

## Author contributions

All authors listed, have made substantial, direct, and intellectual contribution to the work, and approved it for publication.

### Conflict of interest statement

The authors declare that the research was conducted in the absence of any commercial or financial relationships that could be construed as a potential conflict of interest.

## Footnotes

^1^The Spanish education system includes two high school courses, defined by they specialty, for students who plan to attend college. These studies are not binding as basic education, but they are required for university entrance.

## Appendix

Questionnaire

DEMOGRAPHICS Data

Age: ____ Gender: Male Female High school Course:

High school specialty:

High school (name):

Population:

What would you like to study at University?:

What do you know about the University of Castilla-La Mancha?

Would you like to study at the University of Castilla-La Mancha? Yes___ No___

Why?

INSTRUCTIONS:

Enter the website of the University of Castilla-La Mancha: http://www.uclm.es. Enter “Así empezó todo.”

Put over each of the people featured and read the phrase it says. Choose only one of the images, double click, and heard his/her story, then answer the following questions as honestly as possible.

1. Who have you chosen? ______________________________________________________

2. Why have you chosen him/her? _______________________________________________

___________________________________________________________________________

3. How old do you think his/her is? ______________________________________________

4. What did you like the most about his/her has narrated? Why? ________________________

____________________________________________________________________________________________________

5. What did you like the least? Why?

____________________________________________________________________________________________________

6. Has him/her been clearly expressed? Highlights some phrases or comments that have caught your attention.

____________________________________________________________________________________________________

7. Indicates aspects of his/her gestures, look, smile, posture, physical appearance.

Gestures___________________________________________________________Look_____________________

Smile______________________________________________________________________

Position_____________________________________________________________________

Physical appearance___________________________________________________________

8. Has it provided new information about the University of Castilla-La Mancha to you? _________________________________________________________

9. Have you changed your opinion about studying at this University? Yes___ No___

Other observations____________________________________________________________

THANK YOU VERY MUCH FOR YOUR COOPERATION

## References

[B1] Arellano R. (2002). Consumer Behavior, in Focus Latin America. Mexico: McGraw Hill.

[B2] Arnold M. B. (1970). Perennial problems in the field of emotion, in Feelings and Emotions, ed Arnold M. B. (New York, NY: Academic Press), 920–951.

[B3] Association for Research of Media (2015). Navigators in Internet User's Network–Survey. Available online at: www.aimc.es (Accessed December 16, 2015).

[B4] Bagozzi R. Gopinath M. Pieters P. (1999). The ride of emotions in marketing. J. Acad. Mark. Sci. 27, 184–206. 10.1177/0092070399272005

[B5] Barber N. Dodd T. Kolyesnikova N. (2009). Gender differences in information search: implications for retailing. J. Consum. Mark. 26, 415–426. 10.1108/07363760910988238

[B6] Briñol P. Becerra A. Gallardo I. Horcajo J. Valle C. (2004). Validación del pensamiento y persuasión. Psicothema 16, 606–610.

[B7] Briñol P. De la Corte L. Becerra A. (2001). Qué es Persuasión. Madrid: Biblioteca Nueva.

[B8] Burke M. C. Edell J. A. (1989). The impact of feelings on ad-based affect and cognition. J. Mark. Res. 26, 69–83. 10.2307/3172670

[B9] Carrera P. Oceja L. V. (2007). Drawing mixed emotions: Sequential or simultaneous experiences? Cogn. Emot. 21, 422–441. 10.1080/0269993060055790426989980

[B10] Chaiken S. Eagly A. H. (1983). Modalidad de comunicación como determinante de la persuasión. *El papel de relevancia comunicadora*. Rev. Pers. Psicol. Soc. 45, 241–256.

[B11] Chapman D. W. (1981). A model of student college choice. J. Mark. High. Educ. 52, 490–505. 10.2307/1981837

[B12] Chapman R. G. (1986). Toward a theory of college selection: a model of college search and choice behavior. Adv. Consum. Res. 13, 246–250.

[B13] Chapman R. G. (1993). Non simultancous relative performance analysis: Meta- analysis from 80 college-choice surveys with 55, 276 respondents. J. Mark. High. Educ. 4, 405–422. 10.1300/J050v04n01_27

[B14] Clark M. S. Isen A. M. (1982). Toward understanding the relationship between feeling states and social behavior, in Cognitive Social Psychology, eds Hastorf A. H. Icen A. M. (New York, NY: Elsevier), 73–108.

[B15] Coccari R. Javalgi R. (1995). Analysis of students needs in selecting a college or university in a changing environment. J. Mark. High. Educ. 6, 27–39. 10.1300/J050v06n02_0321291107

[B16] Di Muro F. B. Murray K. B. (2012). An arousal regulation explanation of mood effects on consumer choice. J. Consum. Res. 39, 574–584. 10.1086/664040

[B17] Dittmar H. Long K. Meek R. (2004). Buying on the internet: gender differences in on-line and conventional buying motivations. Sex Roles 50, 423–444. 10.1023/B:SERS.0000018896.35251.c7

[B18] Donnellan J. (2002). The Impact of Marketer Controlled Factor Son College-Choice Decisions by Students at a Public Reserch University. Dissertation submitted to the Graduate School of the University of Massachusetts.

[B19] Everhart D. E. Shucard J. L. Quatrin T. Shucard D. W. (2001). Sex-related differences in event-related potentials, face recognition, and facial affect processing in prepubertal children. Neuropsychology 15, 329–341. 10.1037/0894-4105.15.3.329 11499988

[B20] Fernández-Abascal E. G. (1997). Psicología General. Motivación y Emoción. Colección de Psicología. Madrid: Centro de estudios Ramón Areces.

[B21] Fischer E. Arnold S. J. (1994). Sex, gender identity, gender role attitudes, and consumer behavior. Psychol. Mark. 11, 163–182. 10.1002/mar.4220110206

[B22] Gardner H. (1983). Frames of Mind. The Theory of Multiple Intelligences. New York, NY: Basic Books. (Versión castellana (2001). Estructuras de la Mente. La Teoría de las Inteligencias Múltiples. México, FCE).

[B23] Holbrook M. (1986). Aims, concepts, and methods for the representation of individual differences in esthetics responses to design features. J. Consum. Res. 13, 337–347. 10.1086/209073

[B24] Holdsworth D. K. Nind D. (2005). Choice modeling new zealand high school seniors' preferences for university education. J. Market. High. Educ. 15, 81–104. 10.1300/J050v15n02_04

[B25] Hung I. W. Mukhopadhyay A. (2012). Leses of the heart: how actors and observers. perspectives influence emotional experiencies. Int. J. Bus. Soc. Sci. 2, 17.

[B26] Kallio R. (1995). Factors influencing the college choice decisions of graduate students. Res. High. Educ. 36, 109–125. 10.1007/BF02207769

[B27] Keshishian F. McGarr N. S. (2012). Motivating factors influencing choice of major in undergraduates in communication sciences and disorders. Int. J. Speech Lang. Pathol. 14, 174–182. 10.3109/17549507.2011.645250 22390747

[B28] Kim D. Lehto X. Morrison A. (2007). Gender differences in online travel information search: implications for marketing Communications on the Internet. Tourism Management 28, 423–433. 10.1016/j.tourman.2006.04.001

[B29] Kotler P. Kartajaya H. Setiawan I. (2010). Marketing 3.0: From Products to Customers to the Human Spirit. Hoboken, NJ: John Wiley & Sons.

[B30] Kotler P. Keller K. L. (2009). Marketing Management, 13th Edn. Upper Saddle River, NJ: Pearson Prentice Hall.

[B31] Kozinets R. V. De Valck K. Wojnicki A. C. Wilner S. J. S. (2010). Networked narratives: understanding word-of-mouth marketing in online communities. J. Mark. 74, 71–89. 10.1509/jmkg.74.2.71

[B32] Lazarus R. S. (1991). Progress on a cognitive-motivational-relational theory of Emotion. Am. Psychol. 46, 819–834. 10.1037/0003-066X.46.8.819 1928936

[B33] Lim B. C. Chung C. M. Y. (2011). The impact of word-of-mouth communication evaluation on attribute. J. Bus. Res. 64, 18–23. 10.1016/j.jbusres.2009.09.014

[B34] Lin L. (1997). What are student education and educational related institutions? Mark. Res. Today 51, 40–59.

[B35] López B. (2007). Publicidad Emocional: Estrategias Creativas. Madrid: ESIC.

[B36] Marketing Directo (2013). Marketing Directo La Importancia del “Boca a Boca” Para el Éxito de un Producto o una Marca. Available online at: http://www.marketingdirecto.com/actualidad/infografias/la-importancia-del-boca-a-boca-para-el-exito-de-un-producto-o-una-marca/print/ (Accessed March 7, 2016).

[B37] Meyers-Levy J. (1989). Gender differences in information processing: a selectivity interpretation, in Cognitive and Affective Responses to Advertising, eds Cafferata P. Tybout A. M. (Lexington, MA: Lexington Books/DC Heath & Com.), 219–260.

[B38] Meyers-Levy J. Sternthal B. (1991). Gender differences in the use of message cues and judgments. J. Mark. Res. 28, 84–96. 10.2307/3172728

[B39] Meyrowitz J. (1985). Sin Sentido de Lugar. New York, NY: Oxford University Press.

[B40] Mohamad D. Abdullah A. Razali S. Zulkifli S. (2012). Factors influencing students choice to higher institutions: A fuzzy set operation approach. Int. J. Res. Rev. Appl. Sci. 12, 463–467.

[B41] Monroe K. B. Chapman J. D. (1987). Framing effects on buyers' subjective product evaluations. Adv. Consum. Res. 14, 193–197.

[B42] Morales J. F. Rebolloso E. Moya M. (1997). Mensajes persuasivos y cambio de actitudes, in Psicología Social, ed Morales J. F. (Madrid: McGraw Hill), 526–553.

[B43] Murphy P. (1981). Consumer buying roles in college choice: parents and students percepctions. Coll. Univ. 56, 140–150.

[B44] Ohanian R. (1990). Construction and validation of a scale to measure celebrity endorser's perceived expertise, trustworthiness, and attractiveness. J. Advert. 19, 39–52. 10.1080/00913367.1990.10673191

[B45] Peter J. P. Olson J. C. (2010). Consumer Behavior and Marketing Strategy, 9th Edn. New York, NY: McGraw-Hill Irwin.

[B46] Raposo M. Alves H. (2007). A model of university choice: an explotatory approach. Munich Pers. RePEc Archiv. 1, 203–218.

[B47] Rivas J. A. Grande I. S. (2004). Consumer Behavior, in Marketing and Strategy Decisions. Madrid: ESIC.

[B48] Robbins S. (2004). Comportamiento Organizacional. México: Pearson/Prentice Hall.

[B49] Schlecht C. (2003). Celebrities' Impact on Branding. Available online at: http://worldlywriter.com/images/portfolio/proposals/celebrity_branding.pdf (Accessed March 13, 2016).

[B50] Sertoglu A. Catli O. Korkmaz S. (2014). Examining the effect of endorser credibility on the consumers' buying intentions: an empirical study in Turkey. Int. Rev. Manag. Mark. 4, 66–77.

[B51] Shanka T. Quintal V. Taylor R. (2005). Factors influencing international students choice of an education destination- a correspondence analysis. J. Mark. High. Educ. 15, 31–46. 10.1300/J050v15n02_02

[B52] Solomon S. (1998). Psychosocial treatment of posttraumatic stress disorder. Clin. Psychol. 3, 27–41. 10.1002/jclp.10069 12115717

[B53] Soutar G. Turner J. P. (2002). Students preferences for university: a conjoint analysis. Int. J. Educ. Manag. 16, 40–45. 10.1108/09513540210415523

[B54] Taylor S. A. (2009). Reconciling satisfaction, emotions, attitudes, and ambivalence within consumer models of judgment and decision making: A cautionary tale. J. Consum. Satisf. Dissatisf. Complain. Behav. 41–65.

[B55] The ARS Group (2010). Effective Use of Emotions. Available online at: http://segmento.itam.mx/Administrador/Uploader/material/ArticuloEmociones.pdf

[B56] Van der Waldt D. Van Loggerenberg M. Wehmeyer L. (2009). Celebrity endorsements versus created spokespersons in advertising: a survey among students. South Afr. J. Econ. Manag. Sci. 12, 110–114.

[B57] Wachtler J. L. Counselman E. (1981). When increased liking for a comunicator decreases opinión change: an attribution analysis of attractiveness. J. Exp. Soc. Psychol. 117, 197–202. 10.1016/0022-1031(81)90045-7

[B58] Wilkie W. L. (1996). Consumer Behaviour, 3rd Edn. New York, NY: John Wiley & Sons.

[B59] Zajonc R. B. Markus H. (1982). Affective and cognitive factors in preferences. J. Consum. Res. 9, 123–131. 10.1086/208905

[B60] Zhu F. Zhang X. (Michael) (2010). Impact of online consumer reviews on sales: the moderating role of product and consumer characteristics. J. Mark. 74, 133–148. 10.1509/jmkg.74.2.133

